# To Keep the Hands White and Soft

**Published:** 1899-10

**Authors:** 


### To Keep the Hands White and Soft.

In these days of asepsis the hands of the physician, and especially of the surgeon suffer greatly from frequent scrubbings and immersions in antiseptic solutions. A preparation that will keep the hands white and soft and that will not at the same time be inelegant to use, is always in demand. The following formula will be found to be one of the very best ever proposed for this purpose:

| | | |
|---|---|---|
| ℞ | Ol. rosæ . . . . . . . . | gtt. xv |
| | Glycerin . . . . . . . . . . | ʒi |
| | Spts. myrciæ . . . . . . . . | ℥iii |
| | Ol. cajuput . . . . . . . . | gtt. xx |

M. Apply at night before retiring, first washing the hands thoroughly in hot water. In cold weather this can also be applied to the hands before going out.—*The Jour. of the Am. Med-Ass'n.*

